# Adenovirus: ocular manifestations

**Published:** 2020-03-30

**Authors:** Jeremy Hoffman

**Affiliations:** 1 Clinical Research Fellow: International Centre for Eye Health, London School of Hygiene & Tropical Medicine, UK.

**Adenoviral infections of the ocular surface are a common, highly infectious, cause of ocular morbidity. As no specific antiviral agent exists for adenoviral ocular infections, treatment is supportive and robust hygiene measures need to be implemented to reduce its spread; there is no role for topical antibiotics.**

Eye health workers are commonly faced with adenoviral infection of the eye as a cause of bilateral conjunctivitis. Adenoviral infection presents with sudden onset of red eye(s) with a watery discharge often associated with a sore throat. The differential diagnosis includes bacterial (including chlamydial) conjunctivitis, other viral causes of conjunctivitis and, possibly, allergic conjunctivitis. The virus is easily transmitted and there is no specific treatment.

The adenoviruses comprise 51 distinct types of double-stranded DNA viruses that are structurally similar, but antigenically different (serotypes).^1^ These highly stable viruses are found throughout the world and cause respiratory tract, genitourinary, gastrointestinal and ocular infections. Adenoviral infection is usually self-limiting but can lead to fatal multiple-organ failure in immunocompromised individuals.

**“These highly stable viruses are found throughout the world.”**

Adenoviral infection, in common with most other causes of viral conjunctivitis, is highly contagious: the risk of inter-familial infection rates have been estimated at between 10% and 50%.^2^,^3^ The virus can be transmitted via contaminated hands, tissues, towels, from swimming pools as well as medical instruments and devices; possibly also via airborne nasal droplets (from sneezing). Anything that the patient touches (or which touches the patient) can act as a potential source.

To prevent transmission of infection in the eye clinic:

- Always practice good hand hygiene between patients
- Clean used ophthalmic instruments and equipment with disinfectant wipes after each patient.

If adenoviral infection is known or suspected

- If possible, put patients with suspected adenovirus in a separate waiting room. Use a separate examination room if one is available.
- Use disposable gloves
- Avoid contact of the eye with instruments
- If an instrument has come into contact with the eye or hands of a patient or health worker, soak the instrument in a sodium hypochlorite solution for at least 10 minutes before using it again.

## Clinical phenotypes

There are four recognised clinical presentations (phenotypes) for adenoviral conjunctivitis:

**Figure 1:**
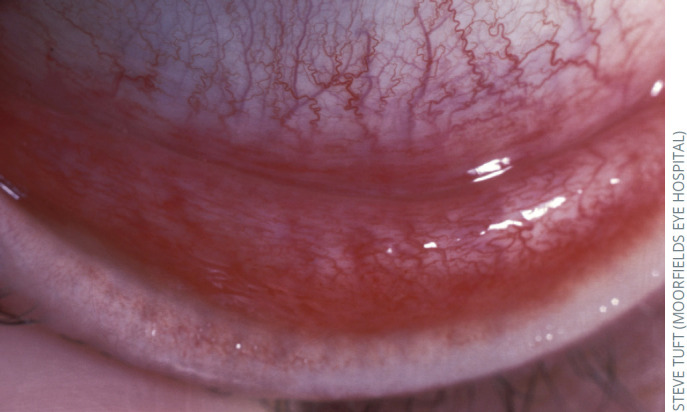
Tarsal conjunctival follicles that are typically seen in viral conjunctivitis.

**Figure 2:**
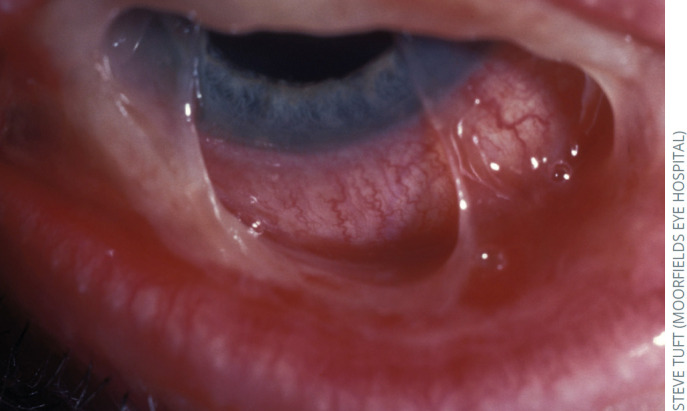
Adenoviral conjunctivitis with conjunctival hyperaemia and significant pseudomembranes. Pseudomembranes can be distinguished from true membranes as they do not bleed when removed. Both forms of membranes must be gently removed by everting the eyelids and using either a cotton-tipped applicator or some sterile forceps to help prevent tarsal scarring and relieve discomfort.

- epidemic keratoconjunctivitis
- pharyngoconjunctival fever
- acute non-specific follicular conjunctivitis
- chronic keratoconjunctivitis.

### Epidemic keratoconjunctivitis (EKC)

Epidemic keratoconjunctivitis (EKC) is the most serious of the adenoviral infections. It is associated with adenovirus serotypes 8 and 19, although associations with other types have been reported.^4^,^5^ Transmission occurs amongst individuals in close contact with one another. It typically affects young adults during autumn and winter, is unilateral in two-thirds of cases, with no systemic features. This is in contrast with pharyngoconjunctival fever (PCF), which is usually bilateral and associated with systemic symptoms of sore throat and fever.

**Figure 3:**
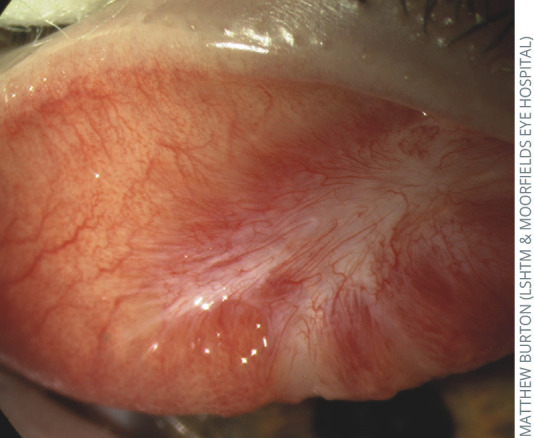
Everted upper eyelid showing tarsal scarring in a patient with a history of membranous adenoviral conjunctivitis. This patient did not have the membranes removed during the acute episode.

After an incubation period of eight days, patients develop significant redness of the conjunctiva, watery discharge from the eye and foreign-body sensation. Signs include tender and swollen lymph nodes in front of the ears (pre-auricular lymphadenopathy, seen in over 50% of cases) together with tarsal conjunctival follicles (Figure 1). The presence of these two signs together is highly suggestive of adenoviral conjunctivitis^6^ and should assist in clinical diagnosis. Other symptoms and signs that may be present include mild photophobia, swelling of the conjunctiva, small subconjunctival haemorrhages, and either pseudo-membranes or true membranes that may bleed when removed (Figure 2). Rarely, symblephara may form and tarsal scarring can occur (Figure 3). If the fellow eye becomes involved, this usually occurs 4–5 days into the disease and the signs are generally less severe than in the first eye, due to partial host immunity.^7^

**Figure 4:**
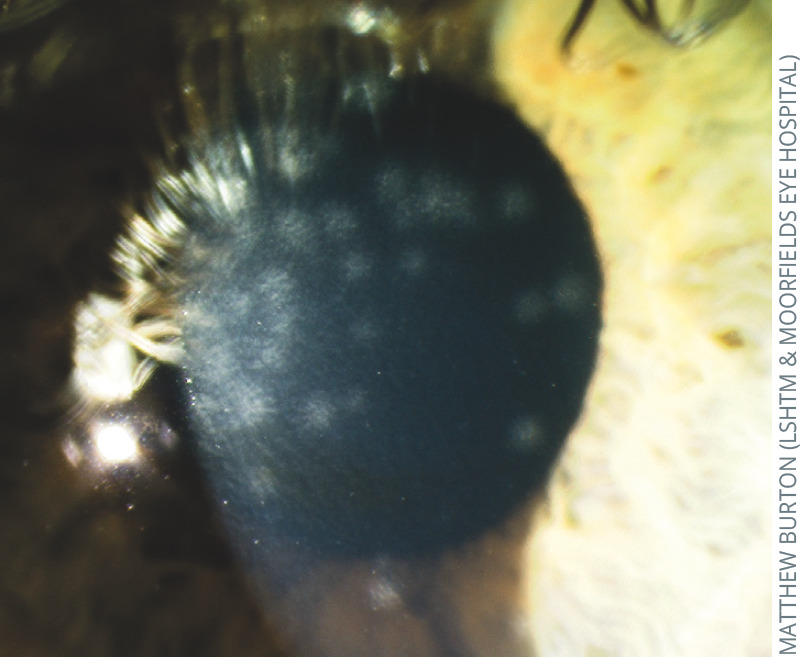
Subepithelial opacities seen in adenoviral keratitis. These typically are non-staining and can persist even after the acute episode of conjunctivitis has resolved.

**Figure 5:**
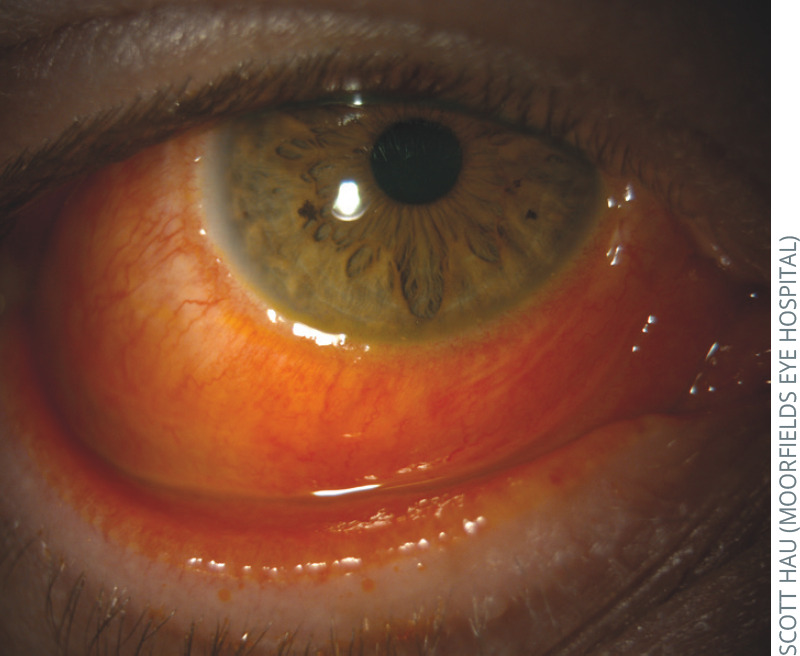
The right eye of a patient with bilateral adenoviral pharyngoconjunctival fever. There is significant conjunctival hyperaemia, petechial subconjunctival haemorrhages and conjunctival follicles.

**Adenoviral keratitis** is divided into four stages (see panel and Figure 4).^4^,^7–9^

**Adenoviral keratitis**

**Stage 1** Diffuse, fine, superficial epithelial punctate keratitis

**Stage 2** Staining focal punctate white epithelial lesions

**Stage 3** Combined epithelial and subepithelial areas

**Stage 4** Non-staining subepithelial macular lesions

- Stage 1 usually occurs within the first week after development of the adenoviral conjunctivitis, with symptoms of worsening discomfort, photophobia and lacrimation. This is due to diffuse superficial epithelial punctate keratitis caused directly by the live virus.
- Stage 2 is characterised by larger, fluorescein-staining white punctate epithelial lesions, and follows soon after Stage 1.
- Stage 3 occurs after a further 24-48 hours with areas of combined epithelial and subepithelial lesions.
- Stage 4 – the adenoviral conjunctivitis has usually started to resolve, and the patient is left with non-staining subepithelial lesions (Figure 4).

Stages 2–4 are thought to develop due to a delayed-type hypersensitivity reaction to the epithelial viral antigens.^7^,^8^

The adenoviral conjunctivitis has usually resolved by two to three weeks after onset, whereas Stage 4 keratitis reaches its peak between weeks three and four. At this point, visual acuity may be reduced by one or two lines. These subepithelial lesions may persist for months and – rarely – years; however, they usually resolve entirely without scarring and visual acuity returns to baseline.

### Pharyngoconjunctival fever

Pharyngoconjunctival fever (PCF) is associated with adenovirus types 3, 4 and 7.^1^,^7^ It is highly contagious and spreads rapidly amongst individuals living or working in close proximity with one another. Unlike EKC, systemic symptoms predominate, with pharyngitis, tender pre-auricular lymphadenopathy, fever and an acute follicular conjunctivitis.

The incubation period is approximately eight days following exposure (range: 5–12 days), when patients develop a high fever with associated muscle pain, malaise and, occasionally, gastrointestinal upset.^1^,^7^ An acute follicular conjunctivitis develops a few days into the symptomatic illness, with initial symptoms of irritation, burning and lacrimation. The conjunctivitis is usually worse in the lower fornix and can lead to a tender, swollen lower eyelid, occasionally with bruising that can mimic orbital trauma. Both eyes are usually involved, although there may be a delay of 1–3 days before the fellow eye becomes symptomatic (Figure 5).

Adenoviral keratitis is less common in pharyngo-conjunctival fever than with EKC but, if present, follows the same progressive four stages as outlined above.

### Acute non-specific follicular conjunctivitis

This form of adenoviral conjunctivitis is caused by numerous adenovirus serotypes. However, it is a mild, self-limiting conjunctivitis commonly seen in children and young adults, which resolves within 7–10 days of symptom onset. Adenoviral conjunctivitis is characterised by soreness and lacrimation, with red conjunctiva and tarsal follicles; the cornea is not involved. The differential diagnosis includes chlamydial or herpes simplex acute follicular conjunctivitis.

### Chronic keratoconjunctivitis

Chronic keratoconjunctivitis caused by adenovirus is rare but has been reported.^10–12^ Clinically, it presents as a relapsing and remitting prolonged course of intermittent conjunctival redness, lacrimation and photophobia. There will be a history of previous viral conjunctivitis within the last 6–9 months. It can be a challenge to diagnose as unlike the other types of adenoviral conjunctivitis, conjunctival papillae rather than follicles predominate. Diagnosis can be confirmed through laboratory investigations as discussed below.

## Investigations

Routine investigations are not usually required for adenoviral conjunctivitis as the diagnosis can be made clinically, particularly if there are conjunctival follicles and tender pre-auricular lymphadenopathy in the absence of significant purulent discharge. When performing investigations for viral conjunctivitis, test for bacterial and chlamydial infection, as well as a range of viruses, including adenovirus and herpes simplex virus.

Available investigations include real-time polymerase chain reaction (RT-PCR), rapid antigen testing, viral culture, and serology for virus-specific IgM and a rise in virus-specific antibody titre over time^7^. Of these, RT-PCR has become the laboratory test of choice as it is rapid with high sensitivity and specificity, although it is not universally available.^13^

Rapid antigen testing has reported sensitivity and specificity of 89% and 94% respectively.^14^ This point-of-care testing, although relatively costly, can be very useful in a primary care setting by preventing the incorrect diagnosis of bacterial conjunctivitis and unnecessary antibiotic use.

## Treatment

There is no specific licensed treatment for adenoviral conjunctivitis. Current advice is targeted at symptom relief with artificial tears and cold compresses to the eyes.^15^ There is no role for antibiotics as they do not protect against secondary infection; unscrupulous use can lead to bacterial resistance and diagnostic confusion as the drops themselves can lead to local toxicity and allergy.^2^,^6^ Beyond these recommendations, treatment is controversial.

The antivirals cidofovir and ganciclovir have been investigated as potential treatments, although the evidence for their routine use is limited.^16–19^

Povidone-iodine (PVP-I) drops is another potential agent for the treatment of adenoviral conjunctivitis^16^. Studies investigating PVP-I in combination with topical steroids (0.1% dexamethasone) have shown a reduction in symptoms and viral titre in both humans and rabbit models.^17^,^18^ A recently published Phase 2 randomised controlled trial suggests some benefit of a combination treatment of PVP-I and dexamethasone in terms of speed of clinical resolution and adenoviral eradication, although this is not yet commercially available and the potential of significant steroid-induced side-effects has not been fully investigated.^19^,^20^

Other than antivirals, there is also controversy surrounding the use of topical steroids to treat adenoviral infection. Although topical steroids can speed the resolution of the signs and relieve symptoms, it is associated with increased viral shedding and duration of active viral infection beyond the typical 12 days, meaning the patient is infectious for longer.^21^,^22^ This can have public health implications, as the number of viruses circulating in the population and environment (the viral reservoir) is increased.

If there is sub-epithelial adenoviral keratitis, topical steroids can rapidly reduce or eliminate the subepithelial opacities, resulting in improved visual acuity. However, on cessation of the topical steroids the opacities usually recur; it is only with time that they will fade.^8^ The established side-effects of topical steroids – raised intraocular pressure and cataract formation – should be considered. However, it is reasonable to treat patients with significant pseudo-membranes or membranes with topical steroids, to reduce the chance of symblephara formation. In addition, patients with significantly reduced vision due to sub-epithelial opacities in the cornea, especially where it is limiting their daily activities, may benefit from treatment – provided they are counselled regarding the side-effects and the potential return of opacities when treatment is stopped.

## Conclusion

Adenoviral ocular infection is highly contagious and usually presents as a follicular conjunctivitis with pre-auricular lymphadenopathy. Although often self-limiting, certain subtypes are associated with a protracted course and significant morbidity; there is also the considerable economic cost to be considered. At present, there is no licensed treatment for this common condition and the treatment currently recommended is supportive, although clinical trials are currently ongoing.
